# Nyamanini Virus Nucleoprotein and Phosphoprotein Organize Viral Inclusion Bodies That Associate with Host Biomolecular Condensates in the Nucleus

**DOI:** 10.3390/ijms24076550

**Published:** 2023-03-31

**Authors:** Yuya Hirai, Masayuki Horie

**Affiliations:** 1 Department of Biology, Osaka Dental University, 8-1 Kuzuha Hanazono-Cho, Hirakata 573-1121, Osaka, Japan; 2 Laboratory of Veterinary Microbiology, Graduate School of Veterinary Science, Osaka Metropolitan University, 1-58 Rinku-Oraikita, Izumisano 598-8531, Osaka, Japan; 3 Osaka International Research Center for Infectious Diseases, Osaka Metropolitan University, Izumisano 598-8531, Osaka, Japan

**Keywords:** Nyamanini virus, non-segmented negative-strand RNA viruses, mononegavirus, nucleoprotein, phosphoprotein, liquid–liquid phase separation, biomolecular condensates, membraneless organelles, nucleus, nuclear speckles

## Abstract

Many mononegaviruses form inclusion bodies (IBs) in infected cells. However, little is known about nuclear IBs formed by mononegaviruses, since only a few lineages of animal-derived mononegaviruses replicate in the nucleus. In this study, we characterized the IBs formed by Nyamanini virus (NYMV), a unique tick-borne mononegavirus undergoing replication in the nucleus. We discovered that NYMV forms IBs, consisting of condensates and puncta of various sizes and morphologies, in the host nucleus. Likewise, we found that the expressions of NYMV nucleoprotein (N) and phosphoprotein (P) alone induce the formation of condensates and puncta in the nucleus, respectively, even though their morphologies are somewhat different from the IBs observed in the actual NYMV-infected cells. In addition, IB-like structures can be reconstructed by co-expressions of NYMV N and P, and localization analyses using a series of truncated mutants of P revealed that the C-terminal 27 amino acid residues of P are important for recruiting P to the condensates formed by N. Furthermore, we found that nuclear speckles, cellular biomolecular condensates, are reorganized and recruited to the IB-like structures formed by the co-expressions of N and P, as well as IBs formed in NYMV-infected cells. These features are unique among mononegaviruses, and our study has contributed to elucidating the replication mechanisms of nuclear-replicating mononegaviruses and the virus–host interactions.

## 1. Introduction

Many mononegaviruses, such as the rabies virus (RABV), measles virus (MeV), respiratory syncytial virus (RSV), and Borna disease virus 1 (BoDV-1), form non-membrane-bound viral biomolecular condensates, called inclusion bodies (IBs), in infected cells. The IBs of mononegaviruses share certain properties, such as their formation via liquid–liquid phase separation, but their morphologies, locations, and components vary among viruses. Viral IBs contain concentrated viral genomes and proteins and thus, are assumed to be the viral replication sites [1,2,3,4], with a few exceptions [5]. Furthermore, some viral IBs sequester proteins involved in the host immune systems and inhibit the innate immune signaling pathway [6,7]. Therefore, studies of viral IBs could provide insights into the basis of viral replication and virus–host interactions.

**Figure 1:**
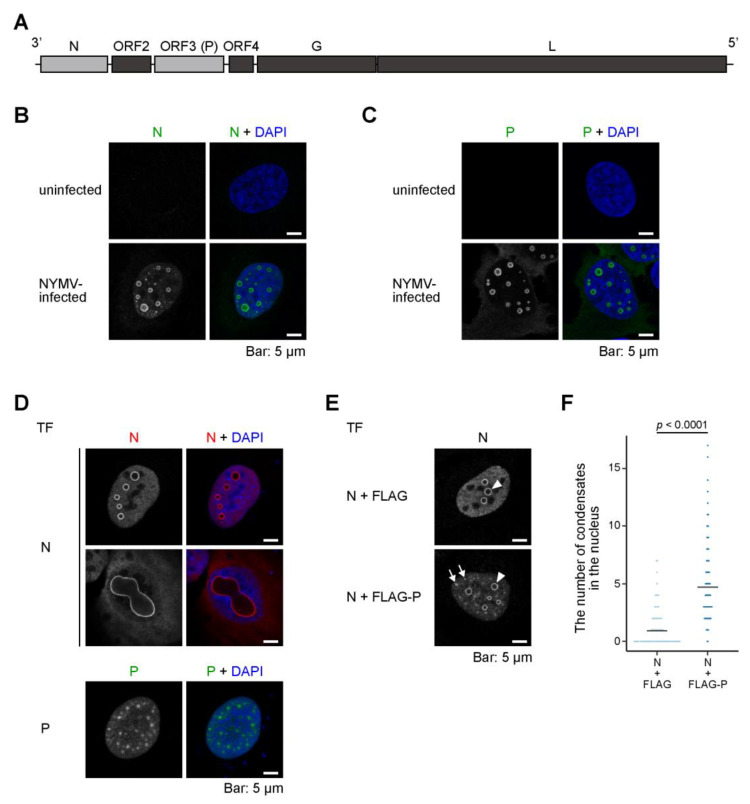
The formation of IBs-like structures by NMYV N and P. (**A**) A schematic diagram of the genome structure of NYMV. The gene regions of N and P are highlighted in grey. (**B**,**C**) The localizations of N (**B**) and P (**C**) in NYMV-infected U-2 OS cells revealed by confocal immunofluorescence microscopy using specific antibodies. (**D**) The localization of N or P that was exogenously expressed by transfection (TF) in uninfected U-2 OS cells revealed by confocal immunofluorescence microscopy using anti-N or anti-P antibodies, respectively. (**E**) The localizations of N in uninfected U-2 OS cells that were transfected with N and FLAG-tag (**upper**) or N and FLAG-tagged P (**lower**). Arrowheads and arrows indicate condensates and puncta, respectively. (**F**) The number of condensates in the nucleus in (**E**). *n* > 300 from three samples. Statistical significance was assessed by using a two-tailed Welch’s *t*-test. The confocal microscopic images presented in (**B**–**E**) were the images of single focal planes.

Nyamanini virus (NYMV) is a tick-borne mononegavirus belonging to the genus *Nyavirus* of the family *Nyamiviridae* [8,9,10,11,12]. The NYMV genome encodes six ORFs (Figure 1A). Based on the sequence similarities of the putative gene products, ORF1 and ORF6 were determined to be nucleoprotein (N) and large RNA-dependent RNA polymerase (L), respectively [11]. ORF5 was predicted to encode a type I transmembrane protein that possesses a signal peptide, suggesting that ORF5 corresponds to glycoprotein (G) [11]. ORF3 was shown to be necessary for the polymerase activity in minireplicon assay, suggesting that ORF3 is a phosphoprotein (P) [13]. ORF4 was demonstrated to be necessary for the formation of the infectious virion and shares certain properties with the matrix protein (M) of other mononegaviruses, suggesting that ORF4 is a counterpart of M [14]. Finally, ORF2 was shown to be necessary for the propagation and formation of virus-like particles; it was also shown to be a negative regulator of the polymerase activity in minireplicon assay, which is analogous to the X protein of BoDV-1 [13,14].

NYMV is unique in that it replicates in the nucleus [13], a feature so far observed only in bornaviruses [15] and Culex tritaeniorhynchus rhabdovirus [16], among animal-derived mononegaviruses. Using antiserum obtained from a mouse infected with NYMV, Herrel et al. showed that NYMV N and P are localized in the host nucleus [13]. Although the details were not provided in their study, IB-like staining signal dots were observed in the nucleus. In addition, Son et al. showed the presence of a double-stranded RNA immunostaining signal in the nucleus, which was co-localized with IB-like staining signal dots, suggesting that the IB-like structures are sites of viral replication [17]. However, to date, the detailed morphology and structure of the dots and the localization of each viral protein remain to be determined. Besides, whether the NYMV proteins affect the host biomolecular condensates in the nucleus remains elusive.

In this study, we characterized the IBs formed by NYMV infection. We revealed that NYMV forms IBs of various structures, from condensates to puncta, in the nucleus, where NYMV N and P are colocalized. We also indicated that both NYMV N and P are required to reconstruct IB-like structures and identified the region of P important for the recruitment of P to the condensates formed by N. Furthermore, nuclear speckles, cellular biomolecular condensates, are reorganized and recruited to the IB-like structures formed by the co-expressions of N and P. These features are unique among mononegaviruses; thus, this study will contribute to a deeper understanding of the replication mechanisms of nuclear-replicating mononegaviruses and the virus–host interactions.

## 2. Results

### 2.1. Both N and P Are Necessary for the Formation of an NYMV-Specific IB-Like Structure

As described above, the nuclear localization of NYMV N and P were shown in a previous study [13], but the detailed subcellular localization of each specific viral protein in NYMV-infected cells has not yet been determined. To examine this, we observed the subcellular localization of N and P in NYMV-infected cells by confocal immunofluorescence microscopy using specific antibodies for each viral protein. We observed nuclear condensates (here, we define ring-like structures in the images of single focal planes as “condensates”) and puncta of various sizes and morphologies in the nucleus, where both N and P were colocalized (Figure 1B,C). These results indicated that NYMV forms various IBs in the nucleus, which contains at least viral N and P.

Afterward, we examined the subcellular localization of N and/or P in cells expressing N alone, P alone, or both N and P. The expression of N alone results in the formation of IB-like condensates in the nucleus (Figure 1D, transfection (TF): N, upper row). Notably, in some cells, considerably larger condensates were present (Figure 1D, TF: N, lower row). On the other hand, when expressing P alone, P formed puncta, but not N-like distinctive condensates, in the nucleus (Figure 1D, TF: P). The co-expression of N and FLAG-tagged P induced the formation of not only condensates seen by the expression of N alone but also numerous puncta (Figure 1E, lower row, arrows) resembling those seen in the NYMV-infected cells (Figure 1B,C). In particular, the number of distinctive condensates increased when co-expressed with FLAG-P (Figure 1F). Together, when expressed individually, NYMV N and P form condensates and puncta in the nucleus, respectively, with morphologies that somewhat differ from the IBs seen in infected cells, while co-expression of both N and P could reconstruct the IB-like structures. Additionally, our data suggest that N and P mutually regulate each other’s localization patterns.

### 2.2. The C-Terminal 27 Amino Acid Residues of P Have the Ability to Be Recruited to the Condensates Formed by N

**Figure 2:**
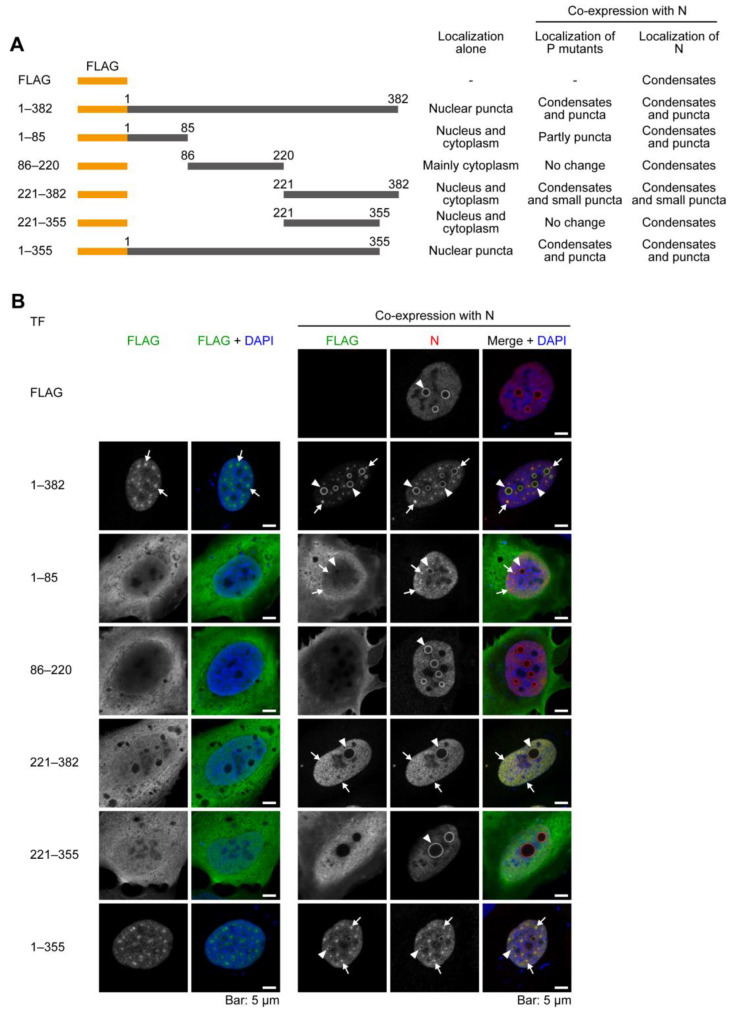
Subcellular localizations of a series of deletion mutants of NYMV P. (**A**) A schematic diagram of a series of deletion mutants of P used in this experiment and the summary of the localizations of P mutants and N when co-expressed with P mutants. Numbers 1–382 represents the full-length P. (**B**) The localizations of a series of deletion mutants of P in uninfected U-2 OS cells in the case of P mutants alone (left column) or when co-expressed with N (right column). Arrowheads and arrows indicate condensates and puncta, respectively. The presented confocal microscopic images were the images of single focal planes.

Previous studies revealed that NYMV N and P interact with each other [13] and that the interaction of N and P is necessary to reconstruct IB-like structures of some mononegaviruses, such as RSV [18] and human parainfluenza virus type 3 [19]. Therefore, we determined which amino acid region of P affects the localization pattern of N and the structures of IB-like condensates and puncta. To do this, we expressed a series of truncated mutants of P (Figure 2A) alone or co-expressed them with N in uninfected cells and observed their localizations. The predicted secondary structure of P is presented in Figure S1. Here, we used FLAG-tagged P mutants due to the lack of anti-N or anti-P antibodies derived from species other than rabbits. The staining patterns of FLAG-P alone or FLAG-P and N are compatible with the above experiments (Figure 2B, 1–382, full-length P), suggesting that the FLAG tag does not affect the localization of P.

FLAG-P_1–85_ alone, which potentially contains the first two helices, did not form remarkable condensates or puncta (Figure 2A, 1–85, left column). However, when FLAG-P_1–85_ was co-expressed with N, N formed not only distinctive condensates (Figure 2A, 1–85, right column, arrowheads) but also puncta (Figure 2A, 1–85, right column, arrows). Although the signals were slight, co-localizations of nuclear condensates and puncta of FLAG-P_1–85_ with N were detected (Figure 2A, 1–85, right column, arrowheads and arrows, respectively). Similarly, FLAG-P_86–220_ alone, which potentially contains a central long helix, did not form remarkable condensates (Figure 2A, 86–220, left column). In particular, when FLAG-P_86–220_ was co-expressed with N, no remarkable changes in the localization patterns of FLAG-P_86–220_ or N were observed. (Figure 2A, 86–220, right column). FLAG-P_221–382_ alone, which potentially contains many coiled regions, also did not form condensates and become localized throughout the cell, as did FLAG-P_1–85_ (Figure 2A, 221–382, left column). However, when FLAG-P_221–382_ was co-expressed with N, FLAG-P_221–382_ was recruited to the nucleus and formed distinctive condensates and many small puncta, which were colocalized with N (Figure 2A, 221–382, right column, arrowheads, and arrows, respectively). Conversely, this nuclear recruitment and the formation of condensates were abolished when the C-terminal 27 amino acids, which potentially contain the last coiled region, were deleted (Figure 2A, 221–355, right column), suggesting that the C-terminal 27 amino acids of P strongly affect the recruitment of the condensates formed by N. On the other hand, the recruitment of P to the condensates formed by N was not only dependent on its C-terminal 27 amino acid residues, but also on the 1–85 amino acid residues because FLAG-P_1–355_ showed a similar localization pattern to that of FLAG-P_1–382_, which formed both condensates and puncta (Figure 2A, 1–355, arrowheads and arrows, respectively). Therefore, these results suggest that the C-terminal 27 amino acid residues of Ps associate with Ns, and the 1–85 amino acid residues may also be important to form viral IB-like structures with N.

### 2.3. Truncated P Mutants Do Not Act as a Polymerase Co-Factor

**Figure 3:**
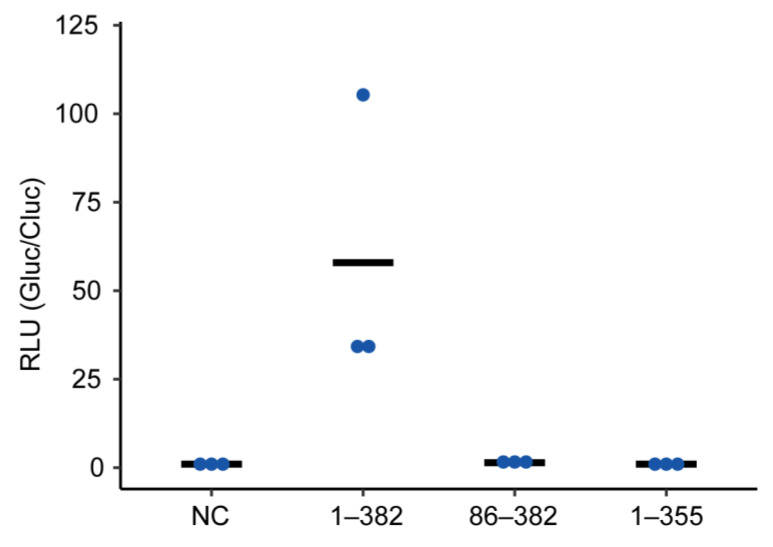
Minireplicon assay using truncated NYMV P. Minireplicon assay was performed on P mutants. The relative luciferase activities are indicated (biological replicates; *n* = 3). The line indicates the average, and blue dots represent individual data. NC, negative control; 1–382, full-length P; 86–382, P_86–382_; 1–355, P_1–355_.

Afterward, we investigated how the amino acid region of P, which is important for the formation of condensates or puncta with N, affects its function as a polymerase co-factor. To do this, we constructed plasmids expressing truncated mutants of P, which lack either the N-terminal (P_86–382_) or the C-terminal region (P_1–355_) important for the association with N, and performed minireplicon assays using the mutants. The results showed that neither P_86–382_ nor P_1–355_ supported the polymerase activity (Figure 3). Thus, this suggests that the formation of condensates or puncta is not necessarily linked to the viral transcription and/or replication.

### 2.4. The Viral IB-like Structures of NYMV Colocalize with Cellular Biomolecular Condensates in the Nucleus

Although many cellular biomolecular condensates are present in the nucleus, the relationship between viral condensates and nuclear biomolecular condensates remains elusive. To examine whether the NYMV viral condensates associate with cellular condensates, we expressed N alone, P alone, or both N and P in uninfected cells and observed their localizations and cell nuclear biomolecular condensates, nucleoli, and nuclear speckles, recognized by anti-B23 and anti-SC-35 antibodies, respectively.

**Figure 4:**
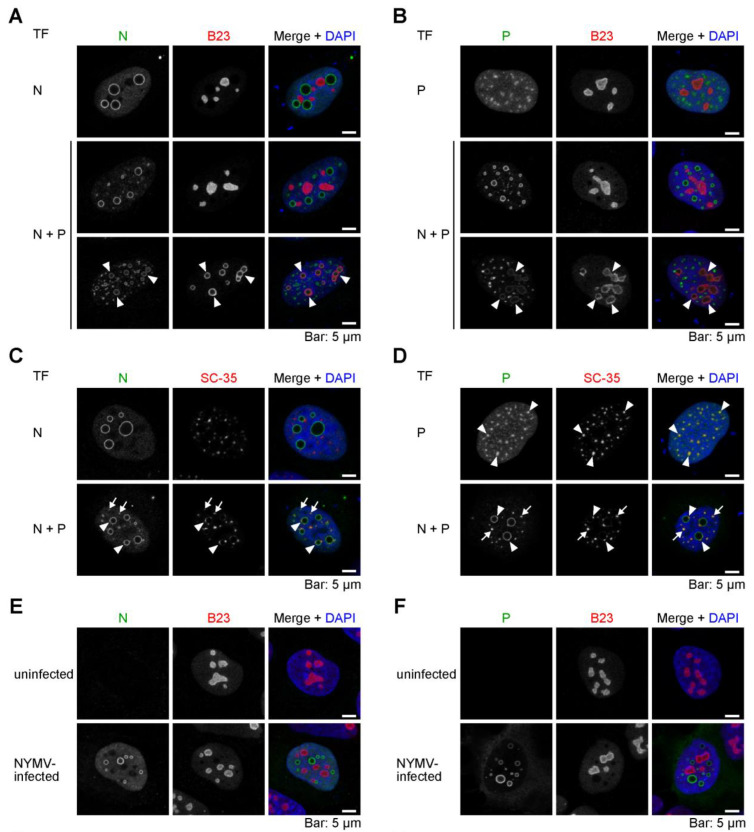

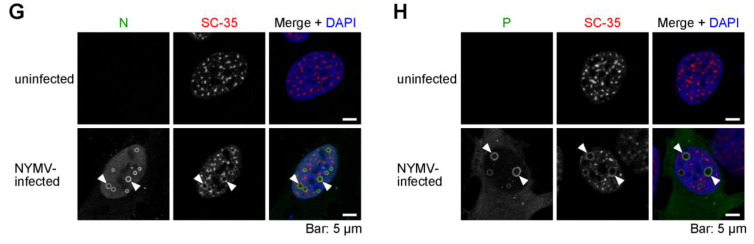
The relationship between viral IB-like structures of NYMV and the nuclear biomolecular condensates. (**A**–**D**) N alone, P alone, or both N and P were expressed in uninfected U-2 OS cells, and their co-localizations with nucleoli recognized by an anti-B23 antibody (**A**,**B**) or nuclear speckles recognized by an anti-SC-35 antibody (**C**,**D**) were examined. Arrowheads indicate abnormal nucleoli co-localized with viral condensates in (**A**,**B**). Arrowheads and arrows indicate nuclear speckle co-localized with viral condensates and puncta, respectively in (**C**,**D**). (**E**–**H**) The co-localizations of N and P with nucleoli (**E**,**F**) or nuclear speckles (**G**,**H**) in NYMV-infected U-2 OS cells were examined. Arrowheads indicate nuclear speckles co-localized with IBs in (**G**,**H**). The presented confocal microscopic images were the images of single focal planes.

When expressing N alone or P alone, we observed that they did not colocalize with nucleoli (Figure 4A, TF: N and Figure 4B, TF: P). Likewise, when co-expressing N and P, neither viral protein colocalized with the nucleoli in most of the transfected cells (Figure 4A,B, TF: N + P, upper row); however, in a few cells, some portion of N and P colocalized with the nucleoli, in which case, the nucleolar structures became abnormal (Figure 4A,B, TF: N + P, lower row, arrowheads).

As for nuclear speckles, N alone did not colocalize with them when expressed (Figure 4C, TF: N), but the expression of P alone resulted in the colocalization of P with them (Figure 4D, TF: P). Furthermore, when co-expressing N and P, nuclear speckles were recruited to both IB-like condensates and puncta (Figure 4C,D, TF: N + P, arrowheads and arrows, respectively).

These tendencies were also observed in NYMV-infected cells. Although nucleoli were not co-localized with IBs in NYMV-infected cells (Figure 4E,F), nuclear speckles were co-localized with the IBs in NYMV-infected cells (Figure 4G,H, arrowheads). Therefore, these results suggest that the viral IB-like structures of NYMV associate with some nuclear biomolecular condensates, particularly with nuclear speckles, where P may serve as a hub of the association.

## 3. Discussion

Many mononegaviruses form remarkable intracellular biomolecular condensates in infected cells, called IBs. Although IBs of mononegaviruses have been studied for many years, their properties, roles in the viral replication cycles, and relationship with host subcellular structures made by individual viruses remain largely unknown. In particular, little is known about the IBs formed in the nucleus due to the rarity of nuclear-replicating animal mononegaviruses. Therefore, in this study, we analyzed the IBs formed by NYMV, a unique mononegavirus that undergoes replication in the host nucleus. We revealed that NYMV forms IBs of various sizes and morphologies in the nucleus. In addition, we showed that NYMV N and P form nuclear condensates and puncta when expressed alone, respectively, and that the co-expression of N and P induced the formation of the intranuclear condensates and puncta morphologically similar to the IBs observed in NYMV-infected cells. Interestingly, we also found that cellular biomolecular condensates were recruited to the IB-like condensates formed by the co-expression of NYMV N and P. To the best of our knowledge, this is the first report showing that a mononegavirus recruits cellular nuclear biomolecular condensates to viral IBs. Thus, this study provides novel insights into the intranuclear condensates, as well as the diverse replication mechanism of mononegaviruses.

The IBs formed by NYMV and BoDV-1, which replicate in the nucleus, exhibit both similarities and differences in their properties. We have previously shown that the IBs of BoDV-1 could be reconstructed by co-expressing N and P [20], which was also observed in NYMV in this study. Further, the morphologies of the distinctive NYMV condensates and BoDV-1 IBs are somewhat similar, in that they both appear as circular structures. However, the expression of either BoDV-1 N or P alone did not result in the formation of any condensates or puncta [20], whereas the expression of NYMV N and P alone induced the formation of condensates and puncta, respectively, despite being morphologically somewhat different from the IBs in NYMV-infected cells (Figure 1). We considered that these differences might be explained by the differences in the structures and biochemical properties of their proteins, such as RNA-binding and oligomerization capacity. Another possible explanation, which is not mutually exclusive with the above reason, is that the variations might arise from the differences in the interaction between each of the viral proteins and the host factors. Indeed, NYMV P apparently associates with nuclear speckles (Figure 4). Therefore, the interaction between NYMV P and nuclear speckles might facilitate the formation of puncta when expressed alone. In the influenza B virus, the viral NS1 protein has been shown to accumulate in nuclear speckles, and it is speculated that its association with nuclear speckles is essential for full viral replication [21]. Considering the function of P as a polymerase co-factor, nuclear speckles (and possibly nucleoli) may be one of the host machineries of interest for elucidating the replication mechanism of NYMV.

Moreover, the above arguments would also be important for understanding the differences among the diverse IBs of mononegaviruses because the minimal components required for the reconstruction of IB-like structures vary among mononegaviruses [5,19,22,23,24,25,26,27,28,29], which may be determined by a combination of various factors, including those described above. Therefore, further studies on the proteins of NYMV and other mononegaviruses and virus–host interactions are needed to understand the diverse IBs of mononegaviruses.

In NYMV, the formation of IBs does not necessarily link to viral transcription and/or replication, although dsRNA was reported to be co-localized with IBs [17]. We showed that none of the truncated mutants of P we examined in the minireplicon assay supported the viral polymerase activity (Figure 3), despite possessing the regions involved in the recruitment of P to the condensates formed by N (Figure 2). Therefore, the P mutants could not act as a polymerase co-factor, despite having the ability to induce IB-like structures together with N. Further studies on the morphologies of IBs and the biochemical and structural features of P would reveal the relationship between the formation of IBs and the viral transcription/replication process.

Our data suggest that both NYMV N and P possess nuclear localization signals (NLSs), as observed in BoDV-1 N [30] and P [31]. When expressed alone, NYMV N and P were predominantly localized in the nucleus (Figure 1 and Figure 2). Although the precise amino acid regions responsible for this localization were not identified, our results indicate that at least one NLS may be present in the 1–355 amino acid region of P, as P_1–355_ was exclusively localized in the nucleus (Figure 2B). Further analyses are required to identify the NLSs of NYMV proteins in order to elucidate the life cycle of nuclear-replicating mononegaviruses.

Taken together, this study provides basic insights into the IBs formed by NYMV, which are unique among mononegaviruses. IBs of mononegaviruses are highly diverse, but the determinants of those differences are still largely unknown. Further studies on IBs would contribute to a deeper understanding of the replication mechanisms of mononegaviruses.

## 4. Materials and Methods

### 4.1. Cells

U-2 osteosarcoma (OS) cells (92022711; European Collection of Authenticated Cell Cultures, Public Health England, London, UK) were cultured in low-glucose (1.0 g/L) Dulbecco’s modified Eagle’s medium (DMEM) (041-29775; Wako or Nacalai Tesque) supplemented with 10% fetal bovine serum (FBS). Vero and 293T cells were maintained in low-glucose DMEM supplemented with 5% and 10% FBS, respectively. The cells were transfected using Lipofectamine 2000^®^ Transfection Reagent (11668027; Thermo Fisher Scientific, Waltham, MA, USA), Polyethylenimine “Max” (24765-1; Polysciences, Inc., Warrington, PA, USA), or Avalanche^®^-Everyday Transfection Reagent (EZT-EVDY-1; Apro Science Group, Tokushima, Japan).

### 4.2. Virus Preparation and Infection

NYMV strain tick 39 was rescued by a reverse genetics system as described previously [14] with slight modifications. First, 293T cells were seeded into a 6-well plate and later transfected with pNYMV (4 μg), pCA-NYMV-N (0.5 μg), pCA-NYMV-P (0.2 μg), and pCA-NYMV-L (0.2 μg). The plasmids were kindly provided by Peter Stäheli. After observing the cytopathic effect, the supernatant was transferred to Vero cells. Then, after 10 days, the supernatant was collected and centrifuged at 2500× *g* for 10 min, and the supernatant was filtered using a 0.22 μm syringe filter, aliquoted to tubes, and stored at −80 °C until use. The virus stock was titrated using Vero cells.

U-2 OS cells (1 × 10^5^) were seeded into 12-well plates with 15 mm coverslips (MATSUNAMI GLASS) and were infected with 7.5 × 10^3^ Tissue Culture Infectious Dose 50 (TCID_50_) of NYMV. After 24 h, the cells were fixed with 4% paraformaldehyde in phosphate buffer (Wako, Osaka, Japan) for 10 min and subjected to immunofluorescence assay (IFA).

### 4.3. DNA Constructs

For the mammalian expression vectors, the coding regions of the N and P genes of NYMV were amplified by polymerase chain reaction (PCR) and then cloned into the pcDNA3 by seamless DNA cloning [32]. The truncation mutants of P (86–382 and 1–355 aa residues) used for minireplicon assay were constructed based on pCAGGS and named as pCA-NYMV-P_86–382_ and pCA-NYMV-P_1–355_.

To construct a Gaussia luciferase (Gluc)-based NYMV minigenome expression vector, the sequences of leader and trailer of NYMV, Gluc, hammerhead ribozyme, and hepatitis delta ribozyme were amplified by PCR and assembled with pCAG-HRSV3 vector (kindly provided by Keizo Tomonaga) using NEBuilder (NEB). The obtained plasmid was designated as pNYMVmg-Gluc.

For bacterial expression vectors, the coding region of the N or P gene was cloned into pET-15b using the NdeI and BamHI sites.

All the primer and plasmid sequences are available upon request.

### 4.4. Antibodies

House-made antibodies against NYMV N and P were obtained as follows. His-tagged recombinant NYMV N (rN) was expressed in Rosetta2 (DE3) at 37 °C for 3 h. The pelleted cells were disrupted by BugBuster Protein Extraction Reagent (Merck Millipore, Burlington, MA, USA), and the inclusion body was purified using BugBuster Protein Extraction Reagent, according to the manufacturer’s instructions. The purified inclusion body was dissolved by 10 mM Tris-HCl pH 7.4, 8 M urea, and then dialyzed against 0.5 M arginine in PBS. His-tagged recombinant NYMV P (rP) was expressed in Rosetta2 (DE3) pLysS at 16 °C for 20 h. The pelleted cells were resuspended in lysis buffer (20 mM Tris-HCl, pH 7.4, 500 mM NaCl, 10 mM imidazole) containing a protease inhibitor cocktail (25955-24; Nacalai Tesque, Kyoto, Japan). After sonication, the lysates were clarified by centrifugation at 20,000× *g* for 10 min at 4 °C. Then, the supernatants were loaded onto a column containing TALON^®^ Metal Affinity Resin (635501; Takara Bio, Kusatsu, Japan). The resins were washed with lysis buffer, and the His-tagged protein was eluted with elution buffer (20 mM Tris-HCl, pH 7.4, 500 mM NaCl, 300 mM imidazole).

The immunization of rabbits with the purified rN or rP and collection of the sera was carried out at Eurofins Genomics.

The following commercial antibodies were also used in this study: anti-FLAG^®^ M2 antibody (F1804; Merck, Burlington, MA, USA), anti-DDDDK-tag pAb (PM020, MBL), anti-B23 antibody (sc-47725, Santa Cruz, Santa Cruz, CA, USA), Monoclonal Anti-Splicing Factor SC-35 (S4045, Merck), goat anti-mouse immunoglobulin G (IgG) (H + L) highly cross-adsorbed secondary antibody, Alexa Fluor 488 (A-11029; Thermo Fisher Scientific), goat anti-mouse IgG (H + L) highly cross-adsorbed secondary antibody, Alexa Fluor 568 (A-11031; Thermo Fisher Scientific), goat anti-rabbit IgG (H + L) highly cross-adsorbed secondary antibody, Alexa Fluor 488 (A-11034; Thermo Fisher Scientific), and goat anti-rabbit IgG (H + L) highly cross-adsorbed secondary antibody, Alexa Fluor 568 (A-11036; Thermo Fisher Scientific).

### 4.5. Immunofluorescence Microscopy

The cells were cultured on coverslips, fixed with 4% paraformaldehyde for 10 min, blocked with 5% bovine serum albumin containing 0.5% Triton X-100 for 15 min, and subsequently probed with primary antibodies for 2 h at RT. Afterward, the cells were washed twice with phosphate-buffered saline (PBS), incubated with the secondary antibodies and 4′,6′-diamidino-2-phenylindole for 1 h at RT, then washed three times with PBS, mounted with ProLong^®^ Diamond Antifade Reagent (P36961, Life Technologies, Carlsbad, CA, USA), and observed using a laser scanning confocal microscope (LSM 700; Carl Zeiss AG, Jena, Germany) equipped with a Plan-Apochromat 63× objective lens (numerical aperture = 1.4).

### 4.6. Minireplicon Assay

pNYMVmg-Gluc (200 ng), pCA-NYMV-N (250 ng), pCA-NYMV-P, pCA-NYMV-P_86–382_, or pCA-NYMV-P_1–355_ (50 ng), pCA-NYMV-L (50 ng), and pSV40-Cluc (10 ng) were transfected to 1.4 × 10^5^ 293T cells in a 24-well plate. After 72 h, the supernatants were collected and subjected to luciferase assays using the Gaussia Luciferase Glow Assay Kit and the Cypridina Luciferase Glow Assay Kit (Thermo Fisher Scientific).

## Data Availability

All data are available within the manuscript.

## Supplementary Materials

The supporting information can be downloaded at: https://www.mdpi.com/article/10.3390/ijms24076550/s1.
