# Supplementary material for: Nyamanini Virus Nucleoprotein and Phosphoprotein Organize Viral Inclusion Bodies That Associate with Host Biomolecular Condensates in the Nucleus
Source: Int J Mol Sci. 2023 Mar 31;24(7):6550. doi: 10.3390/ijms24076550 (PMC10095084; doi:10.3390/ijms24076550)
Supplement: Supplementary file 1 [file ijms-2273669-supplementary.pdf › ijms-2273669-supplementary.pdf]

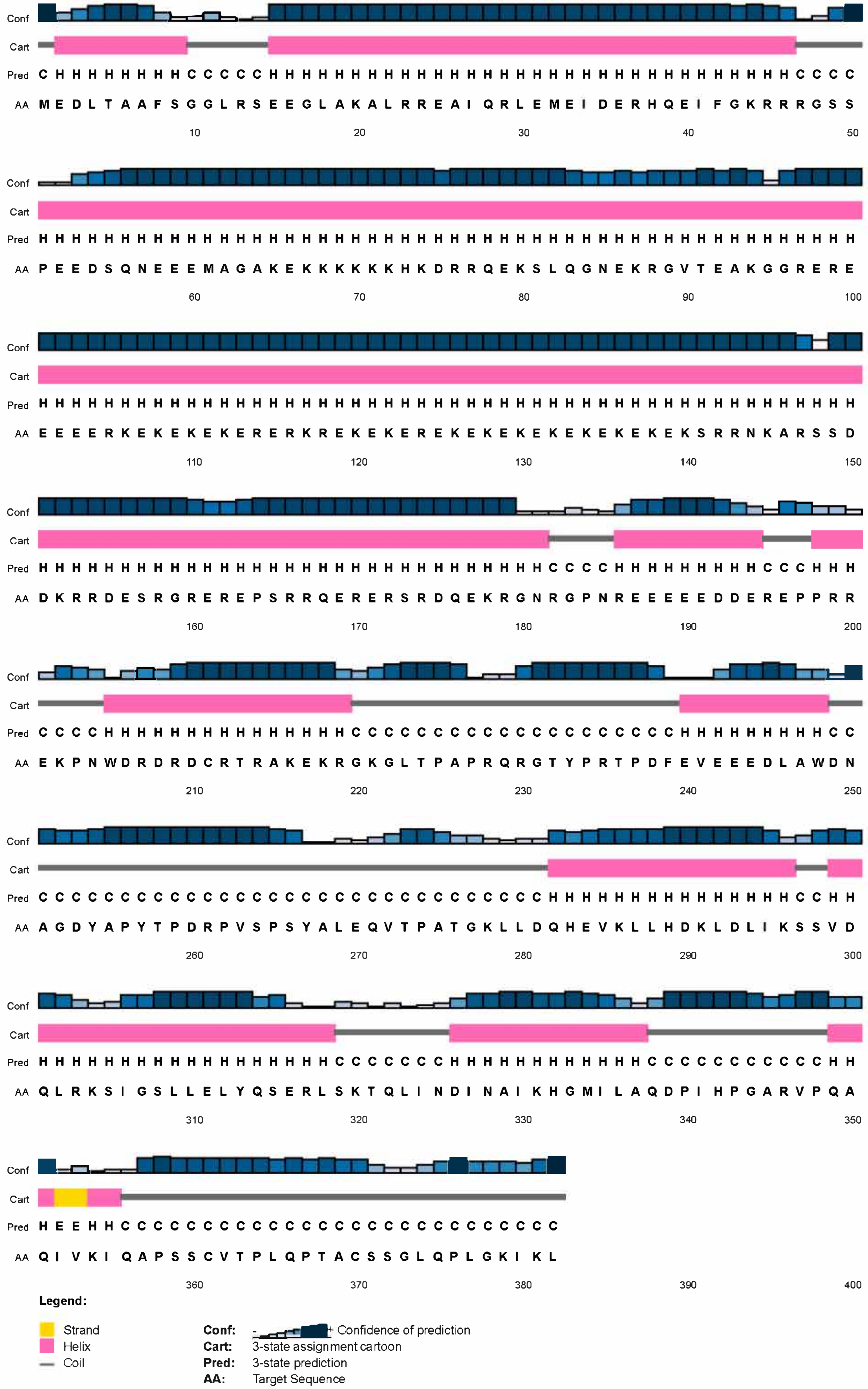

Figure S1. The secondary structure of P predicted by PSIPRED (http://bioinf.cs.ucl.ac.uk/psipred/, accessed on 24 February 2022).
